# Transcriptome sequencing and metabolite analysis for revealing the blue flower formation in waterlily

**DOI:** 10.1186/s12864-016-3226-9

**Published:** 2016-11-09

**Authors:** Qian Wu, Jie Wu, Shan-Shan Li, Hui-Jin Zhang, Cheng-Yong Feng, Dan-Dan Yin, Ru-Yan Wu, Liang-Sheng Wang

**Affiliations:** 1 Key Laboratory of Plant Resources and Beijing Botanical Garden, Institute of Botany, Chinese Academy of Sciences, Beijing, 100093 China; 2 University of Chinese Academy of Sciences, Beijing, 100049 China; 3 College of Horticulture, Nanjing Agricultural University, Nanjing, 210095 China

**Keywords:** Waterlily, Transcriptome, Anthocyanin, Flavonoid, Blue color, *UAGT*

## Abstract

**Background:**

Waterlily (*Nymphaea* spp.), a perennial herbaceous aquatic plant, is divided into two ecological groups: hardy waterlily and tropical waterlily. Although the hardy waterlily has no attractive blue flower cultivar, its adaptability is stronger than tropical waterlily because it can survive a cold winter. Thus, breeding hardy waterlily with real blue flowers has become an important target for breeders. Molecular breeding may be a useful way. However, molecular studies on waterlily are limited due to the lack of sequence data.

**Results:**

In this study, six cDNA libraries generated from the petals of two different coloring stages of blue tropical waterlily cultivar *Nymphaea* ‘King of Siam’ were sequenced using the Illumina HiSeq™ 2500 platform. Each library produced no less than 5.65 Gb clean reads. Subsequently, *de novo* assembly generated 112,485 unigenes, including 26,206 unigenes annotated to seven public protein databases. Then, 127 unigenes could be identified as putative homologues of color-related genes in other species, including 28 up-regulated and 5 down-regulated unigenes. In petals, 16 flavonoids (4 anthocyanins and 12 flavonols) were detected in different contents during the color development due to the different expression levels of color-related genes, and four flavonols were detected in waterlily for the first time. Furthermore, *UA3GT*s were selected as the most important candidates involved in the flavonoid metabolic pathway, *UA3GT*s induced blue petal color formation in *Nymphaea* ‘King of Siam’.

**Conclusions:**

This study will improve our understanding of the molecular mechanism of blue flowers in waterlily and provide the basis for molecular breeding of blue hardy waterlily cultivars.

**Electronic supplementary material:**

The online version of this article (doi:10.1186/s12864-016-3226-9) contains supplementary material, which is available to authorized users.

## Background

Flower color is one of the most important characters of ornamental plants, which determined by many factors. However, the type and content of anthocyanins are generally the primary factor [1, 2]. Although anthocyanins have various structures, there are only seven common chromophore forms (aglycones) of anthocyanins: cyanidin, pelargonidin, delphinidin, peonidin, petunidin, malvidin, and hirsutidin [3, 4]. Among a wide variety of flower colors, blue arises primarily from anthocyanins, which are specifically derived from delphinidin. Breeders always devote to breeding blue cultivars because it is difficult to obtain real blue flowers in many floricultural plants, such as carnation, chrysanthemum, rose, lotus, and hardy waterlily [5, 6]. Traditional breeding methods have many constraints, such as sterility of species and uncontrollable traits, which can be circumvented by genetic engineering [7].

Waterlily (*Nymphaea* spp.), a perennial herbaceous aquatic plant, occurs almost worldwide and has 45-50 species in five subgenera: *Anecphya* (7-10 species), *Brachyceras* (14-16 species), *Hydrocallis* (14 species), *Lotos* (2-3 species), and *Nymphaea* (8 species) [8]. It is called subaqueous nymph. Waterlily has played an obvious role in the history of different cultures, such as ancient Egypt, India, and Thailand. In addition to its ornamental value, waterlily can be used to prepare foods and drinks [9]. It also has diverse biological activities, including antibacterial [10], antimicrobial [11], anti-inflammatory [12], antidiabetic [13, 14], antiradiation [15], antianxiety [16], and antihepatotoxic activities [17]. Waterlily is also divided into two ecological groups, namely, hardy waterlily and tropical waterlily [18]. Hardy waterlily can survive a cold winter and its colors range from dark red to white. Tropical waterlily cannot survive a cold winter in the outdoor pond in the region with the latitude more than 25 degrees, so it has a smaller distribution. However, tropical waterlily is more favorable and it has special flower colors with blue, violet and bluish purple [19]. Thus, breeding hardy waterlily with blue flowers has become an important target for breeders. Although conventional breeding methods may achieve this goal, a truly blue petal color of hardy waterlily has not been reported yet [20].

Molecular breeding provides a new way to obtain blue hardy waterlily. Therefore, it is necessary to understand the molecular mechanism of blue characteristic formation in waterlily and identify related key genes for the purpose of modifying flower color in hardy waterlily by genetic engineering in breeding blue hardy waterlily in the future. The next-generation sequencing technology, RNA sequencing (RNA-Seq), has provided unique insights into the molecular mechanism for non-model organisms without a reference genome [21–23]. As a transcriptome profiling approach utilizing deep-sequencing technologies, RNA-Seq provided the more precise measurements of transcripts and their isoforms than other methods [24].

In the study, the first RNA-Seq project for the petals of two different coloring stages of tropical waterlily cultivar *Nymphaea* ‘King of Siam’ was performed using Illumina HiSeq™ 2500 platform. Qualitative and quantitative analysis of flavonoids were performed with I-Class ultra-high performance liquid chromatography/Xevo triple-quadrupole mass spectrometry (I-Class UPLC/Xevo TQ MS) technology and the expression patterns of differentially expressed genes (DEGs) identified in the flavonoid biosynthetic pathway were analyzed using real-time quantitative polymerase chain reaction (RT-qPCR). The above analysis results can reveal the biochemical and molecular mechanisms of blue color formation in *Nymphaea* ‘King of Siam’ and provide the basis for breeding blue hardy waterlily cultivars. Furthermore, this study will improve our understanding of the molecular mechanism of blue flowers and provide the evidence for molecular breeding theory concerning the basis of flower color modifications in ornamental plants.

## Methods

### Plant materials

**Fig. 1:**
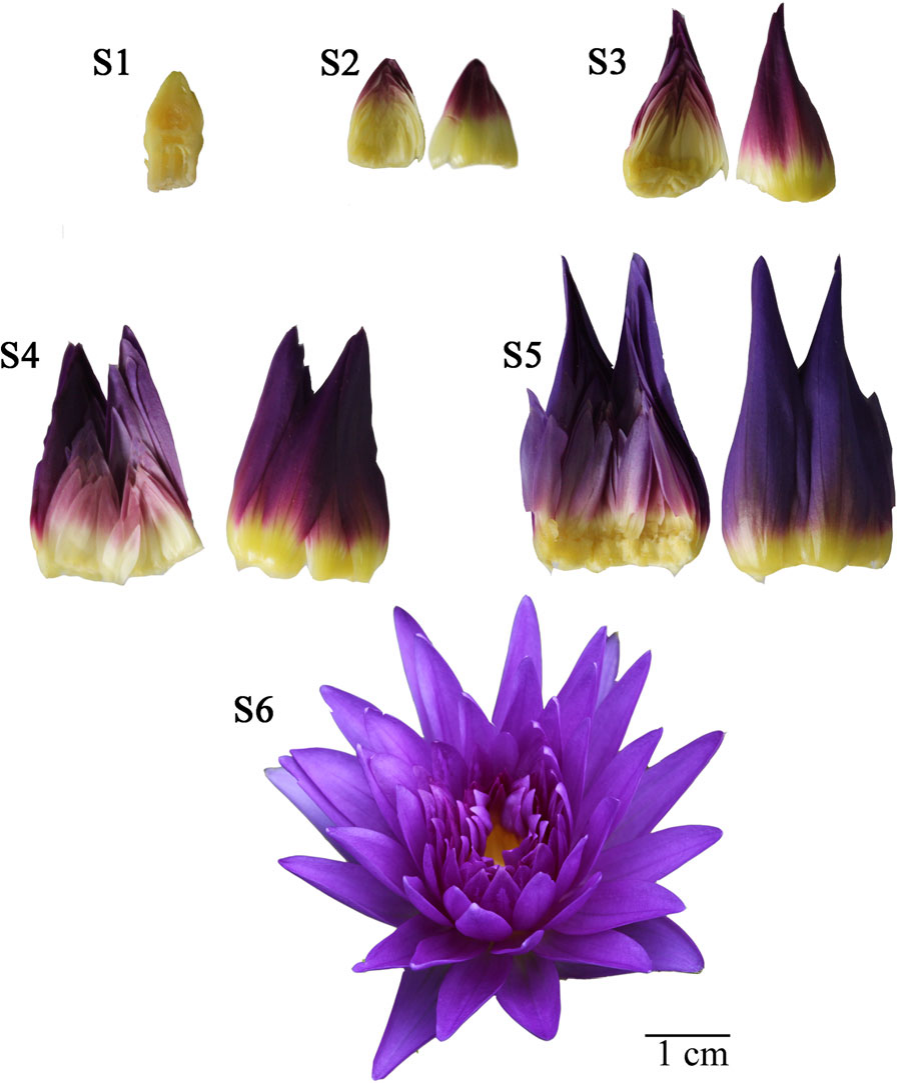
Different development stages of *Nymphaea* ‘King of Siam’. S1, petals were colorless; S2, the outer petals were half colorated; S3, the outer petals were overall colorated while inner petals were colorless; S4, the outer petals were overall colorated while inner petals were half colorated; S5, petals were overall colorated; S6, first day of blooming. Scale bars = 1.0 cm

The petals of tropical waterlily cultivar *Nymphaea* ‘King of Siam’ were selected as the experimental materials. This cultivar has petaloid stamens in deep blue-violet (RHSCC: 92A) color (S6 in Fig. 1). The samples were collected in six different coloring stages in August 2014 at Beijing Botanical Garden, Institute of Botany, the Chinese Academy of Sciences, Beijing, China. In S1, petals were colorless; in S2, the outer petals were partially colorated; in S3, the outer petals were fully colorated, while inner petals were colorless; in S4, the outer petals were fully colorated, while inner petals were partially colorated; in S5, petals were fully colorated; S6 is the first day of blooming (Fig. 1). All the collected petal samples were immediately frozen in liquid nitrogen and stored at −80 °C for RNA extraction and flavonoid analysis.

### Measurement of flavonoids

The extraction of flavonoids was performed according to the previously described method with some modifications [25]. Approximately 0.2 g of frozen petals were pulverized in liquid nitrogen, extracted with 1 mL of extracting solution (99.8: 0.2, v/v, methanol: formic acid) in a test tube, sonicated with KQ-500DE ultrasonic cleaner (Ultrasonic instruments, Jiangsu Kunshan, China) at 20 °C for 20 min, and then centrifuged in SIGMA 3 K30 (SIGMA centrifuger, Germany) with 10000 g for 10 min. The supernatants were collected into fresh tubes. We repeated the above operation for the second time. All extracts were combined and filtered through 0.22 μm reinforced nylon membrane filters (Shanghai ANPEL, Shanghai, China) before the I-Class ultra-high performance liquid chromatography (I-Class UPLC) (Waters, USA) analysis. Three analysis replicates were made for each sample.

I-Class ultra-high performance liquid chromatography/Xevo triple-quadrupole mass spectrometry (I-Class UPLC/Xevo TQ MS) was used for qualitative analysis. The liquid chromatograph was equipped with an ACQUITY UPLC HSS C18 column (2.1 mm × 100 mm, 1.7 μm) (Waters, USA). Eluent A was 1 % formic acid aqueous solution and Eluent B was acetonitrile. The following gradient profile was used: 5 % B at 0 min, 45 % B at 6 min, 90 % B at 7 min, 10 % B at 7.1 min, and 10 % B at 10 min. The flow rate was 0.2 mL/min and the injected volume was 5 μL. Column temperature was maintained at 35 °C and sample temperature was 10 °C. Chromatograms of anthocyanins and other flavonoids were acquired at 525 nm and 350 nm, respectively. The following analysis conditions of mass spectrometry were adopted: the positive-ion (PI) mode for anthocyanins and negative-ion (NI) mode for other flavonoids; capillary voltage of 3.00 kV; cone voltage of 20 V for PI mode and cone voltage of 70 V for NI mode; desolvation gas (N_2_) flow of 800 L/h; cone gas flow of 50 L/h; collision gas flow of 0.12 mL/min; collision energy of 10 eV; the desolvation temperature of 400 °C; source temperature of 150 °C; and the scanning range of 100-1000 (*m/z*) units.

The contents of anthocyanins and other flavonoids were calculated in milligrams per 100 g fresh weight. Cyanidin 3-*O*-glucoside (Cy3Glc) and rutin were used as quantification standards. Mean values and SDs were calculated from three biological replicates.

### RNA isolation, library construction and RNA-Seq

Total RNA was isolated with RN40-EASYspin plant microRNA kit (Aidlab Biotechnology Co., Ltd., Beijing, China), characterized on a 1 % agarose gel and examined with a NanoDrop 2000 UV-vis spectrophotometer (Thermo Fisher Scientific Inc., Waltham, MA, USA). Agilent 2100 Bioanalyzer (Agilent Technologies Co. Ltd., Santa Clara, CA, USA) was used to assess RIN (RNA integrity number) of each sample. The construction of the libraries and RNA-Seq were performed by Biomarker Biotechnology Corporation (Beijing, China). Firstly, mRNA was enriched using Oligo (dT) attached to magnetic beads. Then mRNA was broken into short fragments through the addition of fragmentation buffer. Taking these short fragments as templates, the first-strand cDNA was synthesized with random hexamers. Buffer, dNTPs, RNase H, and DNA polymerase were mixed to synthesize second-strand cDNA. The cDNAs were purified with AMPure XP beads and then subjected to end-repair. To repair cDNAs, an ‘A’ base was inserted at the 3′ end. After that, adaptors were connected to these cDNA fragments to select different fragments. PCR amplification was performed to enrich cDNA library. Agilent 2100 Bioanaylzer and Qubit® Fluorometer (Thermo Fisher Scientific Inc., Waltham, MA, USA) were used to assess the quality of cDNA library. At last, the six libraries were sequenced by using an Illumina HiSeq™ 2500 platform with 125 bp paired-end reads.

### *De novo* assembly and functional annotation

Firstly, after removing the adapter sequences, low quality sequences, unknown reads (N percentage >10 %) and ribosomes RNA from raw reads, the rest of raw reads called clean reads were obtained. Then, clean reads were used for transcriptome *de novo* assembly using trinity platform (http://trinityrnaseq.github.io/) without digital normalization according to the parameters of min_kmer_cov = 2 and other default parameters [26, 27]. Short reads with overlap regions were assembled into longer contigs. Then contigs were linked into transcripts (the length less than 200 bp were filtered) and the transcripts clustered based on nucleotide sequence identity. The longest transcripts in the cluster units were regarded as unigenes to eliminate redundant sequences. These unigenes were annotated using the BLASTx alignment (*E*-value ≤ 10^−5^) to various protein databases, including the NCBI non-redundant (NR) protein database (ftp://ftp.ncbi.nih.gov/blast/db/), the Swiss-Prot protein database (http://www.uniprot.org/), the Kyoto Encyclopedia of Genes and Genomes (KEGG) pathway database (http://www.genome.jp/kegg/), the Clusters of Orthologous Groups of proteins (COG) database (http://www.ncbi.nlm.nih.gov/COG/), the eukaryotic Orthologous Groups of proteins (KOG) database (http://www.ncbi.nlm.nih.gov/COG/), and the Gene Ontology (GO) database (http://www.geneontology.org/). After predicting the amino acid sequences of unigenes by GetOrf (EMBOSS:v6.3.1), we aligned these sequences in Protein family (Pfam, http://pfam.xfam.org/) using HMMER software suite (v3.0) (*E*-value ≤ 10^−10^). The best alignment results from seven databases were chosen to determine the annotation of the unigenes.

### Expression annotation

The alignment package Bowtie (v4.4.7) was used to map reads back to unigenes. According to the comparison results, the expression levels were estimated by means of RSEM (RNA-Seq by expectation maximization) [28, 29]. Unigene expression abundance differences among the samples were represented using Fragments Per Kilobase of transcript per Million mapped reads (FPKM) method [30]. Different expression analysis among different stages was performed with DESeq package (v1.18.0) [31]. Differentially expressed genes (DEGs) were determined based on false discovery rate (FDR < 0.01) and fold change (FC ≥ 2).

### GO and KEGG pathway enrichment analyses for differentially expressed unigenes

GO and KEGG pathway enrichment analyses for the differentially expressed unigenes were then carried out. The obtained GO annotation was enriched and refined using topGo package (v2.12.0) with the “elim” method and Kolmogorov-Smirnov test. KEGG pathways were enriched using in-house scripts according to Fisher’s exact test. Enriched *p*-values were calculated and adjusted with the Bonferroni correction. We selected the corrected *p*-value of 0.05 as the threshold to determine significant enrichment of the gene sets.

### Gene validation and expression analysis

For the purpose of gene validation and expression analysis, all the DEGs related to flavonoid biosynthesis were subjected to real-time quantitative PCR (RT-qPCR) on ABI StepOnePlus™ Real-Time PCR System (Applied Biosystems, USA). Primer sequences designed with Primer Premier 5.0 software are shown in Additional file 1: Table S1. cDNA synthesis and RT-qPCR were performed using FastQuant RT Kit (With gDNase) and SuperReal PreMix Plus (SYBR Green) (TIANGEN Biotech (Beijing) Co., Ltd., China). Actin 11 was selected as an internal control [32]. At least three independent biological replicates for each sample and three technical replicates of each biological replicate were arranged to ensure the reproducibility and reliability of RT-qPCR results.

## Results

### Contents of flavonoids in the petals of *Nymphaea* ‘King of Siam’

To understand the color formations in *Nymphaea* ‘King of Siam’, we compared the metabolic profiles of petals. As expected, anthocyanins were not detected in S1, while four anthocyanins, namely delphinidin 3-*O*-β-galactopyranoside (a1, Dp3Gal), delphinidin 3′-*O*-(2″-*O*-galloyl-β-galactopyranoside (a2, Dp3′galloylGal), delphinidin 3-*O*-(6″-*O*-acetyl-β-glucopyranoside (a3, Dp3acetylGlc), delphinidin 3′-*O*-(2″-*O*-galloyl-6″-*O*-acetyl-β-galactopyranoside (a4, Dp3′galloyl-acetylGal) were detected in other stages. Furthermore, we compared the intermediate products involved in flavonoid biosynthesis. A total of 12 kinds of flavonols, including myricetin derivatives, quercetin derivatives, and kaempferol derivatives were detected in petals. In addition, myricetin 3-*O*-α-L-(3″-*O*-malonyl)-rhamnopyranoside, myricetin 3-*O*-(2″-*O*-galloyl-6″-*O*-malonyl-β-galactopyranoside), quercetin 3-*O*-α-L-(3″-*O*-malonyl)-rhamnopyranoside, and kaempferol 3-*O*-α-L-(3″-*O*-malonyl)-rhamnopyranoside were first detected in waterlily. The retention time, ultraviolet-visible spectral properties and mass spectrometric data, and content of each compound are listed in Additional file 2: Table S2.

**Fig. 2:**
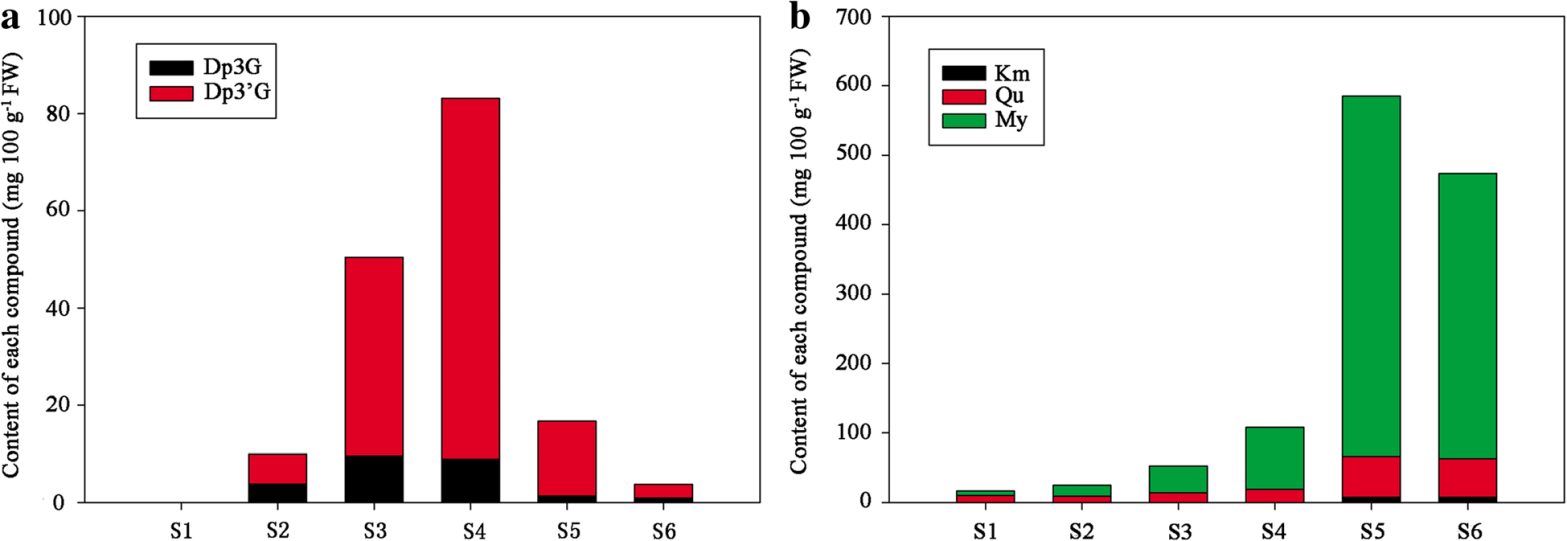
Flavonoid composition obtained by UPLC from six different coloring stages (S1, S2, S3, S4, S5, and S6). Fig. 2a showed the content of anthocyanins. Dp3G contain the content of delphinidin 3-*O*-β-galactopyranoside (Dp3Gal) and delphinidin 3-*O*-(6″-*O*-acetyl-β-glucopyranoside) (Dp3acetylGlc); Dp3′G contain the content of delphinidin 3′-*O*-(2″-*O*-galloyl-β-galactopyranoside) (Dp3′galloylGal) and delphinidin 3′-*O*-(2″-*O*-galloyl-6″-*O*-acetyl-β-galactopyranoside) (Dp3′galloyl-acetylGal). Fig. 2b indicated the content of flavonols. Km contain the content of kaempferol 3-(3″-acetylrhamnoside) and kaempferol 3-*O*-α-L-(3″-*O*-malonyl)-rhamnopyranoside. Qu contain the content of quercetin 7-*O*-galactoside, quercetin 3-*O*-α-L-rhamnopyranoside, quercetin 3-*O*-β-D-(3″-*O*-acetyl)-α-L-rhamnopyranoside and quercetin the content of 3-*O*-α-L-(3″-*O*-malonyl)-rhamnopyranoside. My contain the content of myricetin 3-*O*-β-D-galactopyranoside, myricetin 3-*O*-α-L-rhamnopyranoside, myricetin 3-*O*-α-L-(3″-*O*-acetyl)-rhamnopyranoside, myricetin 3-*O*-α-L-(2″-*O*-acetyl)-rhamnopyranoside, myricetin 3-*O*-α-L-(3″-*O*-malonyl)-rhamnopyranoside and myricetin 3-*O*-(2″-*O*-galloyl-6″-*O*-malonyl-β-galactopyranoside)

These results showed that the content of each composition varied with the coloring stage (Fig. 2). The total anthocyanins content (TA) ranged from 0 to 83.16 mg 100 g^−1^ FW. Dp3′G (including a2 and a4) were the domain anthocyanins and the highest content of Dp3′G (74.26 mg 100 g^−1^ FW) was detected in S4 (Fig. 2a). The total flavonols content (TF) were about 16.41–585.53 mg 100 g^−1^ FW and TF in S5 was nearly five times higher than that in S1 (Fig. 2b). Myricetin derivatives were the main components of flavonols and accounted for approximately 39.01 to 88.67 % of TF, followed by quercetin derivatives (9.84 % of TF in S5 and 61.00 % of TF in S1). The contents of kaempferol derivatives were lower in petals and only detected in S4, S5, and S6 (Additional file 3: Table S3).

### RNA-Seq and *de novo* assembly

To deeply understand the molecular mechanism of blue color formation in waterlily petals, six libraries (S1-1, S1-2, S1-3, S3-1, S3-2, and S3-3) were constructed with blue waterlily cultivar *Nymphaea* ‘King of Siam’ in two different coloring stages and each stage was repeated three times. S1-1, S1-2, and S1-3 were the libraries of the first stage when the petals were colorless; S3-1, S3-2, and S3-3 were the third stage when the outer petals were fully colorated and inner petals were colorless (S1 and S3 in Fig. 1). The six libraries were sequenced by using an Illumina HiSeq™ 2500 platform with 125 paired-end reads (NCBI accession: SRP081259). After cleaning and quality checking, about 36.99 Gb total clean reads were obtained, and each library produced no less than 5.65 Gb clean reads. Q30 percentage (percentage of bases with sequencing error rate lower than 1 ‰) were 90.87 %, 90.93 %, 90.55 %, 90.90 %, 90.78 %, and 90.84 %, respectively. These data showed that the RNA-Seq quality was applicable for further analysis (Additional file 4: Table S4).

Subsequently, *de novo* assembly generated 329,005 transcripts and 112,485 unigenes, with the N50 (covering 50 % of all the nucleotide sequences of the largest unigene length) of 2,418 nt and 1,496 nt. In total, 63,097 unigenes (56.09 %) were between 200 and 500 nt; 38,064 unigenes (33.84 %) were between 500 and 1000 nt; 11,324 unigenes (10.07 %) were longer than 2000 nt (Table 1).

**Table 1:** Length distribution of contigs, transcripts, and unigenes in *Nymphaea* ‘King of Siam’ petals transcriptome

Length Range	Contigs	Transcripts	Unigenes
200-300	19,842,599 (99.50 %)	50,671 (15.40 %)	36,424 (32.38 %)
300-500	37,313 (0.19 %)	45,986 (13.98 %)	26,673 (23.71 %)
500-1000	33,056 (0.17 %)	60,487 (18.38 %)	23,243 (20.66 %)
1000-2000	20,027 (0.10 %)	80,138 (24.36 %)	14,821 (13.18 %)
2000+	10,181 (0.05 %)	91,723 (27.88 %)	11,324 (10.07 %)
Total number	19,943,175	329,005	112,485
Total length	845,487,394	490,152,433	94,130,265
N50 length	43	2,418	1,496
Mean length	42.39	1489.8	836.83

### Functional annotation

**Fig. 3:**
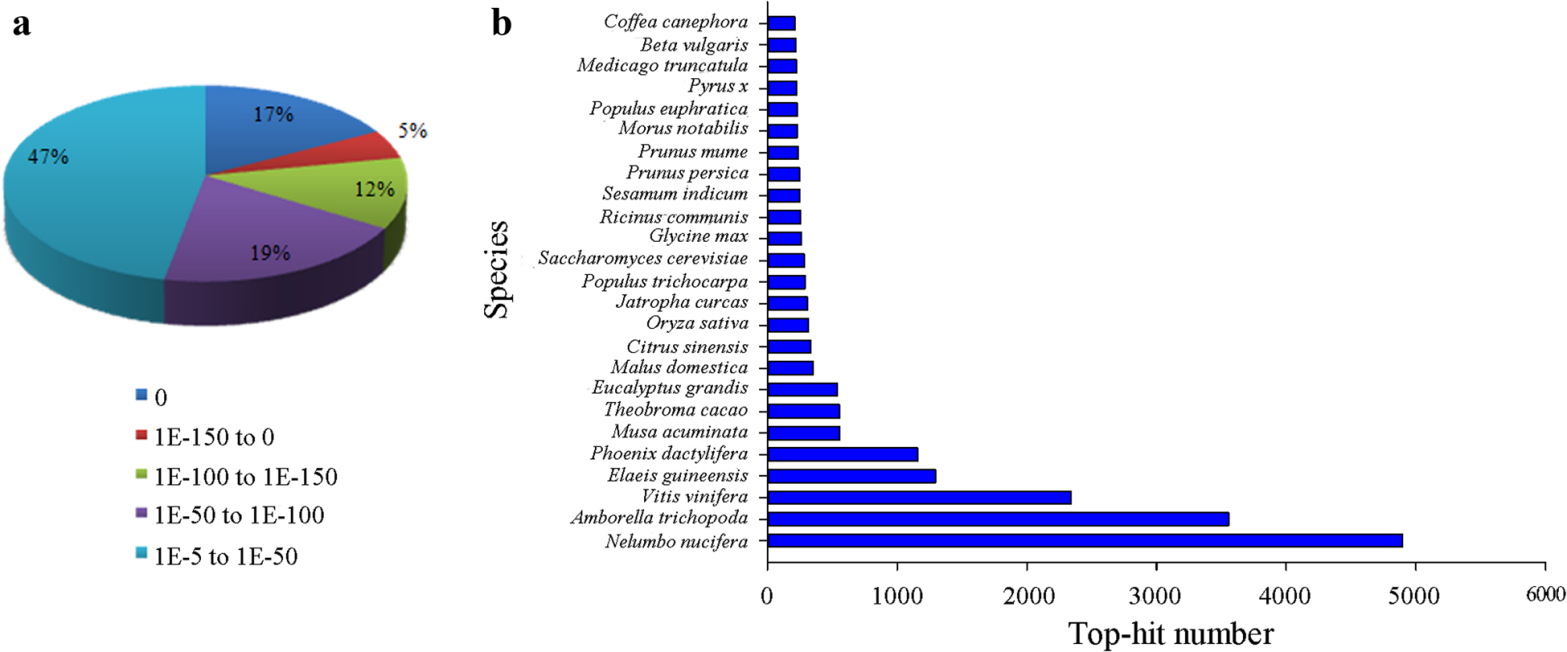
Characteristics of homology search of *Nymphaea* ‘King of Siam’ unigenes. (**a**) *E*-value distribution of the top BLASTx hits against the NR database for each unigene. (**b**) Number and percentage of unigenes matching the top 25 species using BLASTx in the NR database

Due to the lack of a complete genome sequence in *Nymphaea*, only 26,206 unigenes were annotated to seven databases (NR, Swiss-Prot, KOG, COG, GO, KEGG, and Pfam), which accounted for about 23.3 % of 112,485 unigenes. Among them, 25,720 unigenes (98.15 % of all annotated unigenes) could be annotated to the NCBI NR database, while 17,627 (67.26 %), 16,331 (62.32 %) and 15,308 (58.41 %) unigenes could be annotated to Swiss-Prot, Pfam and KOG databases. We annotated 13,526 (51.61 %), 7,968 (30.41 %) and 5,209 (14.39 %) unigenes to GO, COG and KEGG databases. According to the NCBI NR annotation and *E*-value distribution, 53.03 % of the annotated sequences had strong homology (*E*-value < 10^−50^) and 33.75 % of the annotated sequences showed very strong homology (*E*-value < 10^−100^) (Fig. 3a). The 25 top-hit species based on NCBI NR annotation are shown in Fig. 3b. Nearly 50 % of unigenes could be annotated with the sequences from the 5 top-hit species, such as *Nelumbo nucifera*, *Amborella trichopoda*, *Vitis vinifera*, *Elaeis guineensis*, and *Phoenix dactylifera*, while we found that more than 19 % of the sequences showed the highest homology to the sequences from *Nelumbo nucifera*.

17,627 unigenes were annotated against the SwissProt database, accounting for 67.26 % of all annotated unigenes and 121 unigenes that were not annotated against the other three databases. Pfam is the most comprehensive domain annotation of protein structure classification system. In this study, there were 16,331 unigenes noted to Pfam database.

**Fig. 4:**
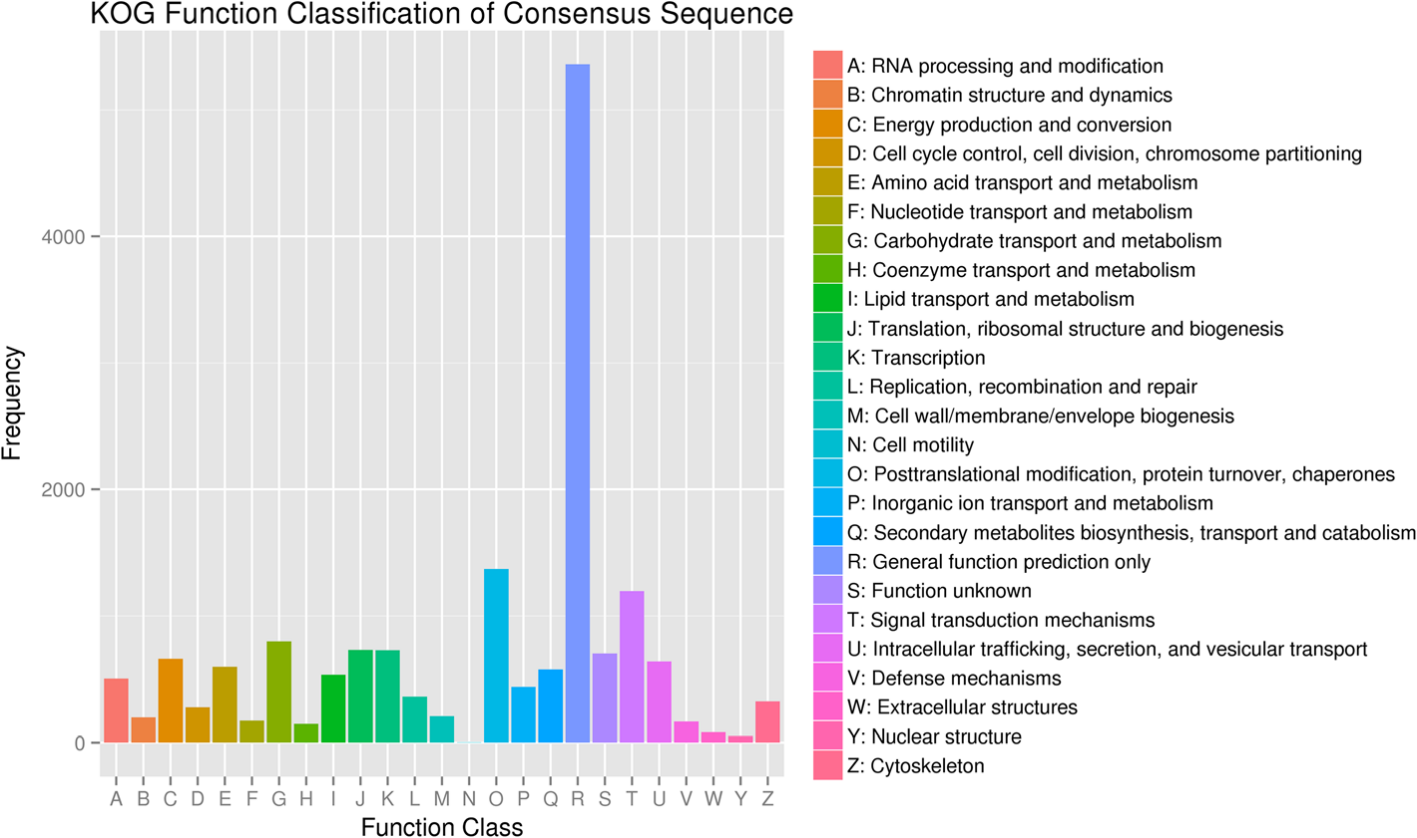
KOG categories of the annotated unigenes

KOG analysis was used to searching orthologous classification and gene evolution rates. There were 15,308 unigenes (58.41 % of all annotated unigenes) that aligned with 25 KOG classifications. Out of the 25 KOG categories, the cluster for general function prediction only (31.86 %) represented the largest group, followed by posttranslational modification, protein turnover, chaperones (8.16 %) and signal transduction mechanisms (7.11 %). Only a small proportion (lower than 1 %) of unigenes was assigned to the number of unigenes which involved in nuclear structure, cell motility and extracellular structure. It is worth noting that a large number of unigenes were assigned to carbohydrate transport and metabolism (4.75 %), translation, ribosomal structure and biogenesis (4.34 %), transcription (4.33 %), energy production and conversion (3.92 %), intracellular trafficking, secretion, and vesicular transport (3.80 %) and secondary metabolites biosynthesis, transport and catabolism (3.43 %) (Fig. 4).

### Identification and GO functional enrichment analysis of DEGs

A total of 1,581 DEGs were obtained comparing S1 and S3 (Additional file 5: Figure S1). Among these, 558 DEGs were assigned one or more GO terms. These DEGs provided clues about the molecular events related to petal development especially petal color development. TopGO package (v2.12.0) was used to map all DEGs in the GO database to exploring the significantly enriched terms compared with the genome backgraound, and corrected *p*-value of 0.05 (Fisher’s exact test) was the threshold. There were a total of 10 enrichment GO terms among three gene ontology categories (Table 2). The results showed that oxidation-reduction process, lipid biosynthetic process, urea transmembrane transport, anthocyanin-containing compound biosynthetic process, pattern specification process and response to light stimulus were the most significant enrichment GO terms under the biological process category. Urea transmembrane transporter activity, peroxidase activity, sequence-specific DNA binding transcription factor activity and heme binding were the most enriched in the molecular function category.

**Table 2:** Gene Ontology functional enrichment analysis of DEGs in *Nymphaea* ‘King of Siam’ petals transcriptome

Gene Ontology term	Cluter frequency	Genome frequency	*P*-value	Corrected *p*-value
Biological Process: oxidation-reduction process (GO:0055114)	59 out of 406, 14.5320197044335 %	773 out of 9953, 7.7665025620416 %	1.65E-06	0.000753462
Biological Process: lipid biosynthetic process (GO:0008610)	6 out of 406, 1.47783251231527 %	15 out of 9953, 0.150708329146991 %	1.62E-05	0.007422674
Biological Process: urea transmembrane transport (GO:0071918)	4 out of 406, 0.985221674876847 %	6 out of 9953, 0.0602833316587963 %	3.83E-05	0.017522614
Biological Process: anthocyanin-containing compound biosynthetic process (GO:0009718)	5 out of 406, 1.23152709359606 %	11 out of 9953, 0.11051944137446 %	4.15E-05	0.018979757
Biological Process: pattern specification process (GO:0007389)	7 out of 406, 1.72413793103448 %	26 out of 9953, 0.261227770521451 %	5.98E-05	0.027344686
Biological Process: response to light stimulus (GO:0009416)	10 out of 406, 2.46305418719212 %	56 out of 9953, 0.562644428815433 %	7.55E-05	0.034521692
Molecular Function: urea transmembrane transporter activity (GO:0015204)	4 out of 437, 0.91533180778032 %	6 out of 10874, 0.0551774875850653 %	3.62E-05	0.009120301
Molecular Function: peroxidase activity (GO:0004601)	11 out of 437, 2.51716247139588 %	69 out of 10874, 0.634541107228251 %	8.64E-05	0.021766185
Molecular Function: sequence-specific DNA binding transcription factor activity (GO:0003700)	17 out of 437, 3.89016018306636 %	155 out of 10874, 1.42541842928085 %	1.60E-04	0.040431117
Molecular Function: heme binding (GO:0020037)	18 out of 437, 4.11899313501144 %	170 out of 10874, 1.56336214824352 %	1.63E-04	0.041003922

### KEGG functional enrichment analysis of DEGs

Pathway analysis is helpful to understand the biological functions and gene interactions. There were 134 DEGs mapped into 63 enriched pathways which contained five main categories while only 4 pathways were selected (*q*-value < 0.05) (Additional file 6: Table S5). The most represented pathways included phenylalanine metabolism (ko00360, 20 DEGs), phenylpropanoid biosynthesis (ko00940, 24 DEGs), followed by flavonoid biosynthesis (ko00941, 21 DEGs) and stilbenoid, diarylheptanoid and gingerol biosynthesis (ko00945, 10 DEGs). From these pathways, information concerning *Nymphaea* ‘King of Siam’ metabolism in two different coloring stages can be obtained. Then, we could understand metabolic pathways more clearly.

### Genes related to color development

**Fig. 5:**
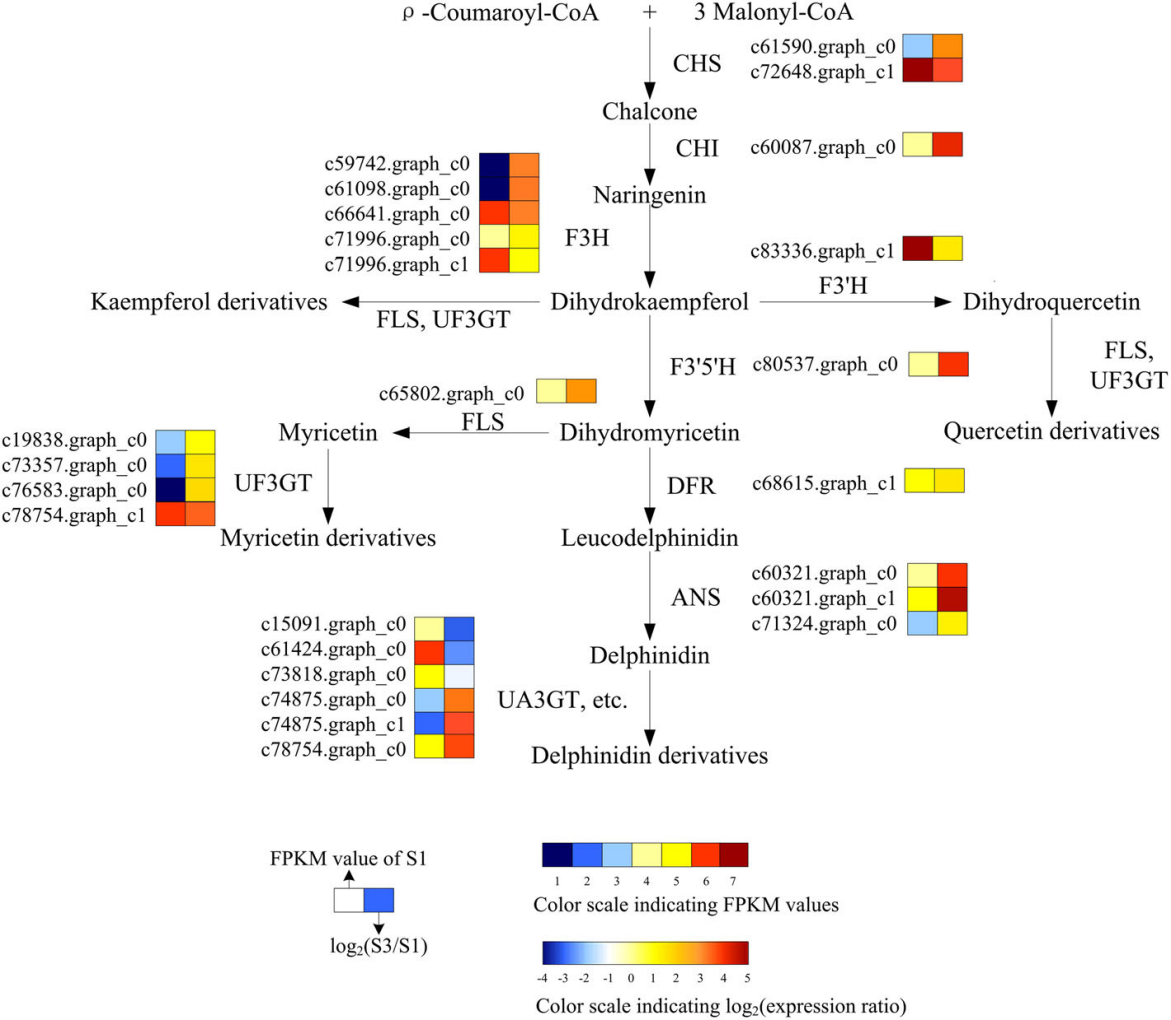
A detailed part of flavonoid metabolic subnetwork showing the subset of nodes or metabolites that constitute the process. Enzyme names, unigene names and expression patterns are indicated at the side of each step. The expression pattern of each unigene is shown on two grids, with the left one representing the FPKM value of S1, and the right one representing the relative log_2_(S3/S1). The grids with seven different colors show the absolute expression magnitude of S1, withV the FPKM values 0–1, 1–2, 2–4, 4–8, 8–16, 16–32, and 32–64 represented by different scale levels 1–7, respectively. CHS: chalcone synthase; CHI: chalcone isomerase; F3H: flavanone 3-hydroxylase; F3′H: flavonoid 3′-hydroxylase; F3′5′H: flavonoid 3′5′-hydroxylase; FLS: flavonol synthase; UF3GT: flavonol 3-*O*-glucosyltransferase; DFR: dihydroflavonol 4-reductase; ANS: anthocyanidin synthase; UA3GT: anthocyanidin 3-*O*-glucosyltransferase

We analyzed the unigenes involved in flavonoid biosynthesis, anthocyanin biosynthesis, and flavone and flavonol biosynthesis pathways in *Nymphaea* ‘King of Siam’ which were related to flower pigmentation. We searched all these genes in the combined functional annotations. A total of 127 unigenes involved in the above three pathways were the study focus (Table 3). Then, we analyzed transcriptional profiles of the genes involved in flavonoid metabolism especially anthocyanin biosynthetic pathway between S1 and S3 to find the key transcripts in the metabolism of blue color. The results demonstrated that a total of 33 key unigenes had significant changes in expression levels, including 28 up-regulated and 5 down-regulated unigenes (Additional file 1: Table S1). All these DEGs contained both upstream genes (*CHS*, *CHI*, etc.) and downstream genes (*UA3GT*, etc.) (Fig. 5). Among them, more than one unigene was annotated as the same enzyme. The formation of blue pigment in waterlily mainly relied on hydroxylation, glycosylation, and acylation rather than methoxylation to maintain its stability, because both the sequences for methoxylation genes involved in anthocyanin modification and the methylated anthocyanin were absent.

**Table 3:** Candidate genes related to flower pigmentation of *Nymphaea* ‘King of Siam’

Function	Gene	Enzyme	ko id (EC no.)	No.All	No. Up	No. Down
Anthocyanin biosynthesis	*CHS*	Chalcone synthase	k00660 (2.3.1.74)	10	2	0
	*CHI*	Chalcone isomerase	k01859 (5.5.1.6)	2	1	0
	*F3H*	Flavanone 3-hydroxylase	k00475 (1.14.11.9)	8	5	0
	*F3′H*	Flavonoid 3′-hydroxylase	k05280 (1.14.13.21)	11	1	0
	*F3′5′H*	Flavonoid 3′,5′-hydroxylase	k13083 (1.14.13.88)	8	1	0
	*DFR*	Dihydroflavonol 4-reductase	k13082 (1.1.1.219)	5	1	0
	*ANS*	Anthocyanidin synthase	k05277 (1.14.11.19)	3	3	0
Anthocyanin modificatioon	*UA3GT*	Anthocyanidin 3-*O*-glucosyltransferase	k12903 (2.4.1.115)	27	3	3
	*UGT75C1*	Anthocyanidin 3-*O*-glucoside 5-*O*-glucosyltransferase	k12338 (2.4.1.298)	4	1	0
	*GT1*	Anthocyanidin 5, 3-*O*-glucosyltransferase	k12938 (2.4.1.-)	7	1	2
	*3GGT*	Anthocyanidin 3-*O*-glucoside 2″-*O*-glucosyltransferase	k12933 (2.4.1.297)	3	0	0
	*3′GT*	Anthocyanidin 3′-*O*-glucosyltransferase	k12939 (2.4.1.238)	6	0	0
	*AA7GT*	Cyanidin 3-*O*-glucoside 5-*O*-glucosyltransferase (acyl-glucose)	k17194 (2.4.1.299)	1	0	0
	*AA5GT*	Cyanidin 3-*O*-glucoside 7-*O*-glucosyltransferase (acyl-glucose)	k17192 (2.4.1.300)	1	0	0
	*5AT*	Anthocyanidin 5-*O*-aromatic acyltransferase	k12936 (2.3.1.153)	1	1	0
Flavone and flavonol biosynthesis	*FNS*	Flavone synthase	k13077 (1.14.11.22)	1	0	0
	*FLS*	Flavonol synthase	k13078 (1.14.11.23)	6	1	0
	*C12RT1*	Flavanone 7-*O*-glucoside 2″-*O*-beta-L-rhamnosyltransferase	k13080 (2.4.1.236)	2	0	0
	*GUSB*	beta-Glucuronidase	k01192 (3.2.1.31)	1	0	0
	*UF3GT*	Flavonol 3-*O*-glucosyltransferase	k10757 (2.4.1.91)	11	4	0
	*FOMT*	Flavonol 3-*O*-methyltransferase	k05279 (2.1.1.76)	5	2	0
	*CROMT2*	Myricetin *O*-methyltransferase	k13272 (2.1.1.149)	1	0	0
Flavanone biosynthesis	*ANR*	Anthocyanidin reductase	k08695 (1.3.1.77)	3	1	0

**Fig. 6:**
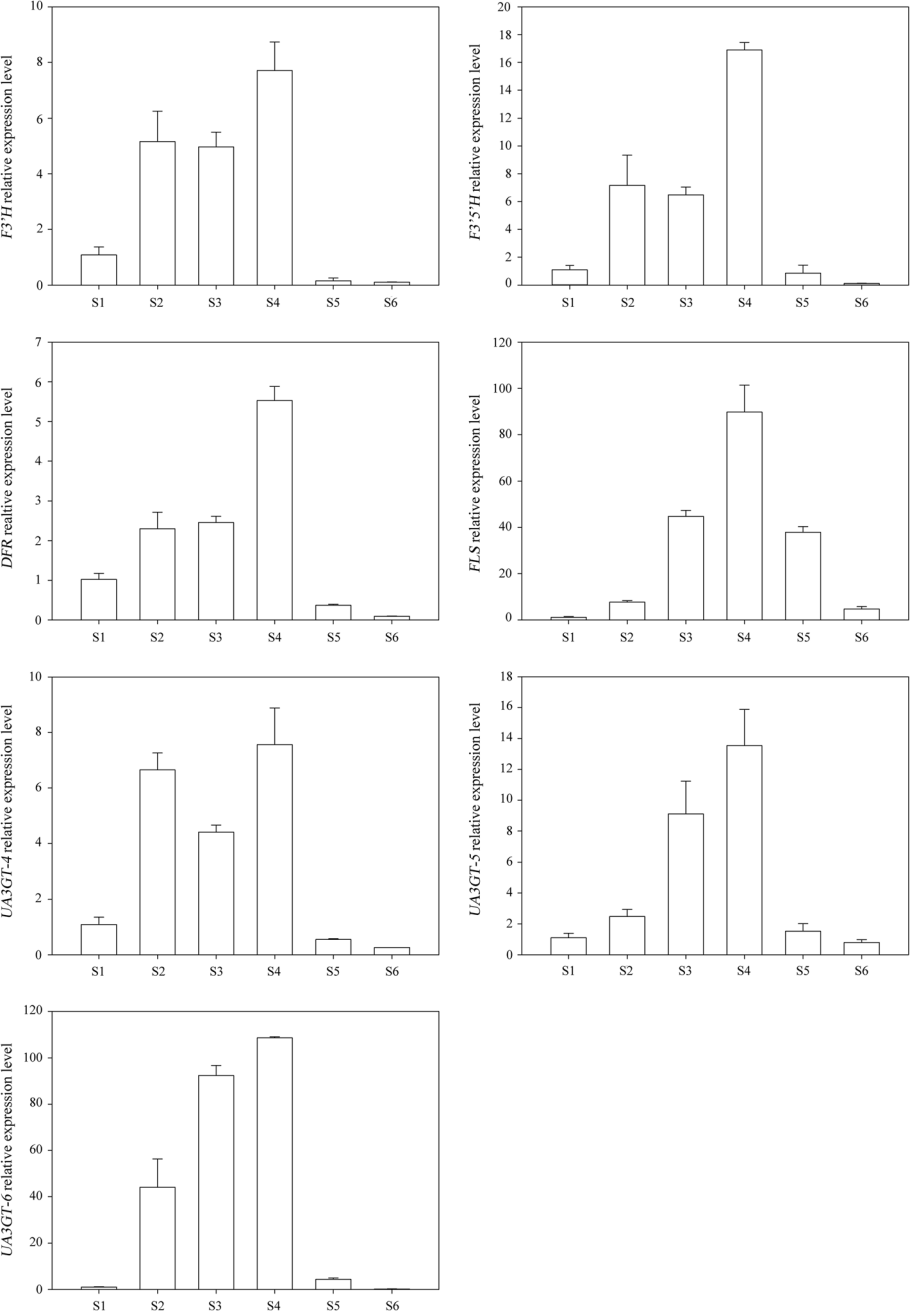
Expression patterns of seven candidate unigenes from six different coloring stages. Actin 11 was used as an internal control. Each unigenes has three biological replicates, and each biological replicate has three technical replicates

In order to verify the credibility of transcriptome sequencing data, the sequences of all these 33 core unigenes, which were not only associated with waterlily color formation but also had different expression levels, were subjected to fluorescent quantitative analysis of RT-qPCR with the designed primers. The results showed that transcriptome sequencing data were credible because the correlation coefficient between transcriptome sequence and RT-qPCR was high (R^2^ = 0.6634) (Additional file 7: Figure S2). Our results of RT-qPCR were almost consistent with those obtained with RNA-Seq method. As shown in Fig. 6, RT-qPCR was also used to study the expression patterns of seven structural unigenes (*F3′H*, *F3′5′H*, *DFR*, *FLS*, *UA3GT-4*, *UA3GT-5*, and *UA3GT-6*) in six different coloring stages. These seven unigenes had similar expression patterns and their expression curves were “bell curves”. With the development of petals, their expression levels increased gradually, reached the maximum level in S4, and then decreased in S5 and S6. *FLS* and *UA3GT-6* had the most rapid change in different stages. In *Nymphaea* ‘King of Siam’, changes in expression of genes encoding pathway enzymes have corresponded to changes in anthocyanin accumulation like many other cases [22, 23, 33, 34].

## Discussion

Waterlily is not only an important ornamental plant in the water, but also have many medicinal effects [35, 36]. Tropical waterlily flowers have the special colors of blue, violet, and bluish purple, while hardy waterlily flowers lack of the above colors. However, the distribution of tropical waterlily is limited because it can only grow in a warm winter, while hardy waterlily is applicable to various grow conditions and requires less labor. For these reasons, breeding blue hardy waterlily can not only satisfy the demand of novel color, but also save cultivation costs. To breed blue hardy waterlily, firstly we should understand the formation mechanism of blue color in tropical waterlily.

Due to the lack of a reference genome sequences, it is difficult to understand the molecular mechanism of blue color formation in tropical waterlily. In the study, we sequenced and comparatively analyzed the transcriptome of the different coloring stages of blue waterlily cultivar *Nymphaea* ‘King of Siam’ petals with the next-generation sequencing technology. A total of 16 flavonoids, including 4 anthocyanins and 12 flavonols were detected in petals of *Nymphaea* ‘King of Siam’. The detected flavonols contained three aglycones, such as myricetin, quercetin, and kaempferol, and the contents of myricetin derivatives were higher. In addition, only delphinidin (Dp) derivatives, such as Dp3Gal, Dp3′galloylGal, Dp3acetylGlc, and Dp3′galloyl-acetylGal were detected. Therefore, we focused on the biosynthesis of Dp derivatives. Among all unigenes involved in the Dp biosynthesis process, two *CHS*, one *CHI*, five *F3H*, one *F3′H*, one *F3′5′H*, one *DFR*, three *ANS*, and three *UA3GT* homologous sequences showed up-regulated expression levels in S3 compared to those in S1. These unigenes covered almost the complete Dp biosynthetic pathway. Blue flower formation was comprehensively regulated by multiple unigenes.

Hydroxylation of flavanones to dihydroflavonols are mediated by F3H, F3′H, or F3′5′H. *F3′H* and *F3′5′H* are called “red gene” and “blue gene” because they are required for Cy and Dp production, respectively. F3′H and F3′5′H are the key enzymes determining the structures of anthocyanins and the color [37]. Many important floricultural crops, such as *Carnations*, *Tulipa*, *Rosa hybrida*, and *Chrysanthemum morifolium*, do not produce Dp and thus lack violet/blue color cultivars. This is attributed to the fact that these crops do not possess the *F3′5′H* gene. Transgenic violet/blue carnations [38], roses [39], and chrysanthemums [40, 41] were developed by expressing a heterologous *F3′5′H* gene. Eight *F3′5′H* were found in *Nymphaea* ‘King of Siam’, whereby one *F3′5′H* expression was up-regulated in S3 (log_2_FC = 4.17). As showed in Fig. 5, *F3′5′H* was crucial in myricetin derivatives and Dp derivatives synthesis. *F3′5′H* expression levels increased from S1, then reached the maximum level in S4, decreased in S5 and S6 (Fig. 6), and the highest total content of myricetin derivatives and Dp derivatives were approximately 535.9 mg 100 g^−1^ FW in S5 (Additional file 3: Table S3). All these analysis indicated that the change of expression level corresponded to the changes in the content of myricetin derivatives and Dp derivatives.

DFR provides one entry step to the biosynthesis of anthocyanins, and can utilized any one or three of the dihydroflavonols (dihydromyricetin, dihydrokaempferol, hyhydroquercetin), resulting in the formation of corresponding leucoanthocyanidins. The three substrates of DFR are similar in the structure and only different in the number of hydroxyl groups on the B phenyl ring, which is not the site of enzymatic action. It is not surprising that DFRs from many plant species can utilize all three dihydroflavonols. However, in some species, DFR has strict substrate specificity [42]. In this study, pelargonidin or cyanidin-based anthocyanins have not been detected. There are two possible explanations for this. Firstly, DFRs in *Nymphaea* ‘King of Siam’ only utilize dihydromyricetin. Secondly, DFRs utilize dihydromyricetin, dihydroquercetin, and dihydrokaempferol, but FLS can utilize dihydroquercetin and dihydrokaempferolin priority. The competitiveness of *DFR*/*FLS* was significant to *Nymphaea* ‘King of Siam’ because it could change both content of flavonols and anthocyanins. In this study, expression level of *FLS* was higher than *DFR*. Therefore, the contents of flavonols were higher than anthocyanins. As co-pigment, flavonols played an important role in color formation. Change the expression level of *DFR*/*FLS* may lead to varying degrees to change the color in waterlily.

Anthocyanins are modified at one or several positions by methylation, glycosylation, or acylation. These modifications endow anthocyanins with unique properties, including color shade, stability, and solubility [43]. Methylation is not as important as glycosylation or acylation, because methylated anthocyanin were not detected in the petals of *Nymphaea* ‘King of Siam’, and the sequences of methoxylation genes involved in anthocyanin modification were not obtained in assembled results. Metabolites of anthocyanins had been compared in previous studies [25, 44–47]. In tropical waterlily, delphindin 3′-glycosides were the main anthocyanins in blue cultivars; delphindin 3′-glycosides and delphindin 3-glycosides were detected in blue/violet ones; only delphindin 3-glycosides were found in violet/red petals. In red cultivars, cyanidin 3-glycosides and delphindin 3-glycosides were detected both in red tropical and hardy waterlily. The determined contents of anthocyanins in white, yellow, and pink cultivars were low or zero, indicating that the structural modification (especially 3′-position modification) by down-stream genes was crucial to blue color formation. Therefore, 3′-glycosyltransferases (3′GTs) might play an important role in blue petal color formation of waterlily. 3′GTs provide the basis of 3′-polyacylations. Two 3′GT have been identified: GtUA3′GlcT of *Gentiana triflora* and anthocyanin 3′, 5′-glucosyltransferase (CtUA3′5′GlcT) of *Clitoria ternatea* [7, 48, 49].

Six *UA3′GT* homologous unigenes were found in waterlily (Additional file 8: Table S6). However, their FPKM were very low, expression levels had no significant change between S1 and S3. For this reason, these six UA3′GT may be not the key enzymes for blue formation in waterlily. Different from other species, waterlily may have other unigenes which can also catalyse 3′-position glycosides. Kogawa et al. [49] found that UA3′5′GT was important in *Clitoria ternatea* which accumulated polyacylated anthocyanins named ternatins, UA3′5′GT could be glycoslated at 3′-, 5′-position of delphindin. Ogata et al. [50] had reported that RhGT1 could be glycosylated at two different sites (3-, 5-position) of cyanidin in roses. In this study, we compared the expression levels and patterns of all glycosyltransferase in anthocyanin biosynthetic pathway and found that *UA3GT*s might be the most important enzymes for the accumulation of delphindin 3′-glycosides which are the main anthocyanins in blue petals.

*UA3GT*s were selected as candidates involved in flavonoid metabolic pathway, *UA3GT*s induced blue color petals in *Nymphaea* ‘King of Siam’. All these results revealed the biochemical and molecular mechanisms of blue color formation and provided the basis for breeding blue hardy waterlily cultivars. This study will improve our understanding of the molecular mechanism of blue flowers and provide the evidence for molecular breeding theory concerning the basis of flower color modifications in ornamental plants.

## Conclusions

The transcript dataset derived by using high-throughput sequencing technology provided an efficient approach for identifying vital unigenes in *Nymphaea*. Transcriptome analyses from the petals of two different coloring stages of blue tropical waterlily cultivar *Nymphaea* ‘King of Siam’ resulted in 112,485 unigenes. 127 unigenes could be identified as putative homologues of color-related genes in other species, including 28 up-regulated and 5 down-regulated unigenes. The composition of flavonoids and the metabolic pathways related to blue formation were identified, and *UA3GT*s were selected as the most important candidates involved in the flavonoid metabolic pathway, which induced blue petal color formation. This study provides a comprehensive molecular biology background for researches on *Nymphaea*, particularly with respect to the process of blue color formation.

## Additional files

Additional file 1: Table S1. List of DEGs which related to flavonoid biosynthesis in flower petals of *Nymphaea* ‘King of Siam’. (DOCX 25 kb)
Additional file 2: Table S2. Qualitative analysis of flavonoids in flower petals of *Nymphaea* ‘King of Siam’. (DOCX 21 kb)
Additional file 3: Table S3. Quantitative analysis of flavonoids in flower petals of *Nymphaea* ‘King of Siam’. (DOCX 18 kb)
Additional file 4: Table S4. Output statistics of RNA-Seq of *Nymphaea* ‘King of Siam’. (DOCX 17 kb)
Additional file 5: Figure S1. Volcano plot between S1 and S3. (PNG 28 kb)
Additional file 6: Table S5. List of pathway enriched differentially expressed genes in *Nymphaea* ‘King of Siam’. (DOCX 16 kb)
Additional file 7: Figure S2. Correlation of gene expression results respectively obtained by two methods (RT-qPCR analysis and RNA-Seq) for color-related genes in S1 and S3. (TIF 1641 kb)
Additional file 8: Table S6. List of *UA3′GT* in *Nymphaea* ‘King of Siam’. (DOCX 17 kb)

## Abbreviations

ANS: Anthocyanidin synthase
CHI: Chalcone isomerase
CHS: Chalcone synthase
COG: Clusters of Orthologous Groups of proteins
Cy: Cyanidin
Cy3Glc: Cyanidin 3-*O*-glucoside
DEGs: Differentially expressed genes
DFR: Dihydroflavonol 4-reductase
Dp: Delphinidin
Dp3′galloyl-acetylGal: Delphinidin 3′-*O*-(-2″-*O*-galloyl-6″-*O*-acetyl-β-galactopyranoside
Dp3′galloylGal: Delphinidin 3′-*O*-(-2″-*O*-galloyl-β-galactopyranoside
Dp3acetylGlc: Delphinidin 3-*O*-(-6″-*O*-acetyl-β-glucopyranoside
Dp3Gal: Delphinidin 3-*O*-β-galactopyranoside
F3′5′H: Flavonoid 3′, 5′-hydroxylase
F3′H: Flavonoid 3′-hydroxylase
F3H: Flavanone 3-hydroxylase
FLS: Flavonol synthase
GO: Gene Ontology
I-Class UPLC/Xevo TQ MS: I-Class ultra-high performance liquid chromatography/Xevo triple-quadrupole mass spectrometry
KEGG: Kyoto Encyclopedia of Genes and Genomes
KOG: Eukaryotic Orthologous Groups of proteins
N50: Covering 50 % of all the nucleotide sequences of the largest unigene length
NR: Non-redundant
Pfam: Protein family
Q30 percentage: Percentage of bases with sequencing error rate lower than 1‰
RNA-Seq: RNA sequencing
RT-qPCR: Real-time quantitative polymerase chain reaction
TA: Total anthocyanins content
TF: Total flavonols content
UA3GT: UDP-glycoside: Anthocyanin 3-*O*-glycosyltransferase
