# Supplementary material for: Transcriptome sequencing and metabolite analysis for revealing the blue flower formation in waterlily
Source: BMC Genomics. 2016 Nov 9;17:897. doi: 10.1186/s12864-016-3226-9 (PMC5101690; doi:10.1186/s12864-016-3226-9)
Supplement: Additional file 1: Table S1. — List of DEGs which related to flavonoid biosynthesis in flower petals of Nymphaea ‘King of Siam’. (DOCX 25 kb) [file 12864_2016_3226_MOESM1_ESM.docx]

**Additional Table S1. List of DEGs which related to flavonoid biosynthesis in flower petals of *Nymphaea* ‘King of Siam’.**

| **Gene** | **Unigene id** | **FDR** | **Log_2_FC** | **Reference annotation** | **Forward primer sequence (5'–3')** | **Reverse primer sequence (5'–3')** |
| --- | --- | --- | --- | --- | --- | --- |
| *CHS* | c61590.graph_c0 | 1.32E-13 | 2.20 | Chalcone synthase GN=CHS1 OS=Casuarina glauca (Swamp oak) PE=2 SV=1 | ATGTGGCCGTACTCGCTGA | CATTGGGCATCTCCGACTGG |
|  | c72648.graph_c1 | 6.24E-142 | 2.85 | Chalcone synthase GN=CHS1 OS=Casuarina glauca (Swamp oak) PE=2 SV=1 | CAGGATGGTGAGCGTTGAGG | CTTGTGCTCGCTGTTGGTGA |
| *CHI* | c60087.graph_c0 | 4.10E-211 | 4.28 | Chalcone-flavonone isomerase 2 GN=CHI2 OS=Vitis vinifera (Grape) PE=3 SV=1 | GGAAGGACAGCGTTAGTGCC | AAGTCTTCCCTCCTGGTTCCTC |
| *F3H* | c59742.graph_c0 | 1.96E-09 | 3.32 | Naringenin, 2-oxoglutarate 3-dioxygenase GN=F24M12.280 OS=Arabidopsis thaliana (Mouse-ear cress) PE=1 SV=1 | CGACGGTGCCTTTGTCATCA | AGTGGGTAGACAAGGGCATCA |
|  | c61098.graph_c0 | 4.20E-10 | 3.39 | Flavanone 3-dioxygenase GN=FHT OS=Petroselinum crispum (Parsley) PE=1 SV=1 | AGCGTTCCCTTCCTACACTGAG | AACGATGTTGCGGAAGACG |
|  | c66641.graph_c0 | 3.41E-192 | 3.32 | Naringenin, 2-oxoglutarate 3-dioxygenase (Fragment) GN=AN3 OS=Petunia hybrida (Petunia) PE=1 SV=1 | CTGGCCTGAGCTGGAAGCA | GCCTCCCAGCCATACATAACC |
|  | c71996.graph_c0 | 3.40E-06 | 1.27 | Naringenin, 2-oxoglutarate 3-dioxygenase OS=Hordeum vulgare (Barley) PE=2 SV=1 | TATGCAGACAGTTTGTTGCCAGT | TCGTTGTCCCTTCACGCTTT |
|  | c71996.graph_c1 | 1.17E-13 | 1.10 | Flavanone 3-dioxygenase GN=FHT OS=Petroselinum crispum (Parsley) PE=1 SV=1 | GCAAGCAGGAAGCAAAGAAA | ATCCACCTCATTCCTATACCAAGC |
| *F3'H* | c83336.graph_c0 | 1.53E-62 | 1.70 | Flavonoid 3’-monooxygenase GN=CYP75B2 OS=Petunia hybrida (Petunia) PE=2 SV=1 | CAATGCGTGGGAGGGAAAG | GCAAGCACGGTGGAATGG |
| *F3'5'H* | c80537.graph_c0 | 1.20E-261 | 4.12 | Flavonoid 3’, 5’-hydroxylase 2 GN=CYP75A3 OS=Petunia hybrida (Petunia) PE=2 SV=1 | TCAAGAGGCTTCTCCCAAACG | GGAGACTGGAGGAATCGGACAT |
| *DFR* | c68615.graph_c1 | 2.31E-43 | 1.54 | Dihydroflavonol 4-reductase GN=DFR OS=Vitis vinifera (Grape) PE=1 SV=1 | GCTGCTTCTCCTGGCAACAC | TGACCTGTTGCGGAGGAAGT |
| *ANS* | c60321.graph_c0 | 4.45E-165 | 4.11 | Leucoanthocyanidin dioxygenase GN=ANS OS=Malus domestica (Apple) PE=2 SV=1 | CTGTGCCAACTTCTATCGCCC | GGAAATAATAGGGCGCTTTGTT |
|  | c60321.graph_c1 | 0 | 4.83 | Leucoanthocyanidin dioxygenase GN=ANS OS=Malus domestica (Apple) PE=2 SV=1 | CGGAAAGAGGAGGAAGCAGG | AAACGCCAAACAGTGGGAAA |
|  | c71324.graph_c0 | 7.61E-11 | 1.35 | 1-aminocyclopropane-1-carboxylate oxidase homolog 10 GN=At5g43450 OS=Arabidopsis thaliana (Mouse-ear cress) PE=2 SV=1 | AAGGCGTAACTCGCTGGGA | GCGGGAGATGGAGACTGTTG |
| *FLS* | c65802.graph_c0 | 5.54E-09 | 2.11 | Flavonol synthase/flavanone 3-hydroxylase GN=FLS OS=Citrus unshiu (Satsuma mandarin) PE=1 SV=1 | TGCCTCCTGGTAGCATTAGCA | CGACGCTATGGACTGAACCC |
| *UF3GT* | c19838.graph_c0 | 0.007935883 | 1.06 | UDP-glycosyltransferase 74F2-like [Cucumis sativus] | CGCCCGATGGAGTGAGTTG | CAGCACTTAGGAAGGCACAGC |
|  | c73357.graph_c0 | 1.14E-08 | 1.55 | 7-deoxyloganetin glucosyltransferase GN=UGT85A24 OS=Gardenia jasminoides (Cape jasmine) PE=1 SV=1 | GGAAGTAACACCAGAAGCCACAG | AGCCTCCTCATCCTCAACCC |
|  | c76583.graph_c0 | 0.002748023 | 1.80 | UDP-glycosyltransferase 74D1 GN=F20M17.21 OS=Arabidopsis thaliana (Mouse-ear cress) PE=1 SV=1 | GGTAGGCGAGGAGGAAGACG | CCATCATTTGTTCCTCAGTCCC |
|  | c78754.graph_c1 | 2.04E-135 | 2.61 | UDP-glucose flavonoid 3-*O*-glucosyltransferase 7, partial [Manihot esculenta] | CCGTCGTCTACCTCAGTTTC | GGTTTGCTTCTCGGGCTTCA |
| *FOMT* | c70707.graph_c0 | 0.001829395 | 1.34 | Caffeic acid 3-*O*-methyltransferase GN=COMT1 OS=Catharanthus roseus (Madagascar periwinkle) PE=2 SV=1 | CAACAACCCAGACGCACCA | CATATCGCCTTTGGACTTTGC |
|  | c76615.graph_c0 | 2.62E-11 | 1.22 | Trans-resveratrol di-*O*-methyltransferase GN=ROMT OS=Vitis vinifera (Grape) PE=1 SV=2 | GCTCCGCATAGCAGACATCAT | ATGCACCAGTAGGCGCATTAG |
| *UA3GT* | c15091.graph_c0 | 1.29E-30 | -3.00 | Anthocyanidin 3-*O*-glucosyltransferase GN=RT OS=Petunia hybrida (Petunia) PE=2 SV=1 | CTGGCAACGGCAGAACAACT | ACCCACAATGGCTGACGAAC |
|  | c61424.graph_c0 | 1.37E-20 | -2.50 | Putative UDP-glucose flavonoid 3-*O*-glucosyltransferase 3 OS=Fragaria ananassa (Strawberry) PE=2 SV=1 | CGAGGTGAGGAAGGTGGAGC | CACCGTTTCTTCCTTCATCGTC |
|  | c73818.graph_c0 | 8.74E-08 | -1.09 | Anthocyanidin 3-*O*-glucosyltransferase GN=RT OS=Petunia hybrida (Petunia) PE=2 SV=1 | AGGTTCGTGCTCGGCAGATA | ACTTCATCTACCACTGGCTCCC |
|  | c74875.graph_c0 | 1.68E-19 | 2.40 | Putative UDP-glucose flavonoid 3-*O*-glucosyltransferase 3 OS=Fragaria ananassa (Strawberry) PE=2 SV=1 | GCGTCTCATTGCGTTTGTGC | CCCTCGCCCTTCTACCCTC |
|  | c74875.graph_c1 | 2.66E-09 | 2.83 | Putative UDP-glucose flavonoid 3-*O*-glucosyltransferase 3 OS=Fragaria ananassa (Strawberry) PE=2 SV=1 | CGCCCTCCACTCATCTTCCT | CGGCCTTCGTTGTTGGTATG |
|  | c78754.graph_c0 | 6.33E-79 | 3.87 | UDP-glycosyltransferase 78D4 GN=UGT78D4 OS=Arabidopsis thaliana (Mouse-ear cress) PE=2 SV=2 | TCACCATCTCCACCAACGC | CACTGTGAGGGATGGGATTTT |
| *UGT75C1* | c74699.graph_c0 | 2.05E-16 | 1.16 | Crocetin glucosyltransferase, chloroplastic (Precursor) GN=UGT75L6 OS=Gardenia jasminoides (Cape jasmine) PE=1 SV=1 | AGGGAAGGTTCTAATGCCAATCA | CGACGATTTGATGGCAGTGTA |
| *GT1* | c55884.graph_c0 | 4.04E-20 | 1.45 | Putative UDP-glucose flavonoid 3-*O*-glucosyltransferase 3 OS=Fragaria ananassa (Strawberry) PE=2 SV=1 | TGGCTCCGAAGCGAAGAAT | CCGAAGCACAAGAACAGAACG |
|  | c59089.graph_c0 | 0.006891062 | -2.26 | Anthocyanidin 5,3-*O*-glucosyltransferase GN=RhGT1 OS=Rosa hybrid cultivar PE=2 SV=1 | TTGCTGGTGAAGGAGATGGG | CTCGCTCCCTCAACCCAGA |
|  | c77357.graph_c0 | 5.51E-08 | -2.31 | UDP-glycosyltransferase 88A1 GN=UGT88A1 OS=Arabidopsis thaliana (Mouse-ear cress) PE=2 SV=1 | CACGGGTGAGCCCAGGTT | AAGTCCTCTACCATCCTAGCCATT |
| *5AT* | c77214.graph_c0 | 3.88E-06 | 1.76 | BAHD acyltransferase DCR GN=MRO11.2 OS=Arabidopsis thaliana (Mouse-ear cress) PE=2 SV=1 | GCTTTCTCAAATCCCACCTCCT | CAGCAGAGCGACCAAGTATTCAC |
| *ANR* | c62277.graph_c0 | 8.00E-12 | 1.02 | Anthocyanidin reductase GN=T13M11.8 OS=Arabidopsis thaliana (Mouse-ear cress) PE=1 SV=2 | GTGTTACAGCGGCAGCAGAG | CGGTGCGCTTCGATTCTAC |
| *Actin 11* |  |  |  |  | ATGTGGCACTGGACTATGAGC | AGAGTTGTAAGTGGTTTCGTGAAT |
