# Supplementary material for: Transcriptome sequencing and metabolite analysis for revealing the blue flower formation in waterlily
Source: BMC Genomics. 2016 Nov 9;17:897. doi: 10.1186/s12864-016-3226-9 (PMC5101690; doi:10.1186/s12864-016-3226-9)
Supplement: Additional file 2: Table S2. — Qualitative analysis of flavonoids in flower petals of Nymphaea ‘King of Siam’. (DOCX 21 kb) [file 12864_2016_3226_MOESM2_ESM.docx]

**Additional Table S2. Qualitative analysis of flavonoids in flower petals of *Nymphaea* ‘King of Siam’.**

| **compound** | **Rt (min)** | **λmax (nm)** | **EST-PI MS/MS^2^ (m/z)** | **EST-NI MS/MS^2^ (*m/z*)** | **Identifacation** |
| --- | --- | --- | --- | --- | --- |
| a1 | 3.35 | 277, 525 | 303[A+H]^+^, 465[M+H]^+^ |  | delphinidin 3-*O*-β-galactopyranoside |
| a2 | 3.5 | 276, 528 | 303[A+H]^+^, 617[M+H]^+^ |  | delphinidin 3’-*O*-(2”-*O*-galloyl-β-galactopyranoside) |
| a3 | 4.02 | 276, 527 | 303[A+H]^+^, 507[M+H]^+^ |  | delphinidin 3-*O*-(6”-*O*-acetyl-β-glucopyranoside) |
| a4 | 4.25 | 277, 529 | 303[A+H]^+^, 659[M+H]^+^ |  | delphinidin 3’-*O*-(2”-*O*-galloyl-6”-*O*-acetyl-β-galactopyranoside) |
| f1 | 4.59 | 277, 347 | 319[A+H]^+^, 481[M+H]^+^ | 316[A-2H]^−^, 479[M-H] ^−^ | myricetin 3-*O*-β-D-galactopyranoside |
| f2 | 4.93 | 262, 350 | 319[A+H]^+^, 465[M+H]^+^ | 316[A-2H] ^−^, 463[M-H] ^−^ | myricetin 3-*O*-α-L-rhamnopyranoside |
| f3 | 5.03 | 253, 367 | 303[A+H]^+^, 465[M+H]^+^ | 301[A-H] ^−^, 463[M-H] ^−^ | quercetin 7-*O*-galactoside |
| f4 | 5.46 | 255, 346 | 303[A+H]^+^, 449[M+H]^+^ | 300[A-2H] ^−^, 447[M-H] ^−^ | quercetin 3-*O*-α-L-rhamnopyranoside |
| f5 | 5.65 | 261, 348 | 319[A+H]^+^, 507[M+H]^+^ | 316[A-2H] ^−^, 505[M-H] ^−^ | myricetin 3-*O*-α-L-(3”-*O*-acetyl)-rhamnopyranoside |
| f6 | 5.87 | 261, 348 | 319[A+H]^+^, 507[M+H]^+^ | 316[A-2H] ^−^, 505[M-H] ^−^ | myricetin 3-*O*-α-L-(2”-*O*-acetyl)-rhamnopyranoside |
| f7 | 6.24 | 255, 348 | 303[A+H]^+^，491[M+H]^+^ | 300[A-2H] ^−^，489[M-H] ^−^ | quercetin 3-*O*-β-D-(3”-*O*-acetyl)-α-L-rhamnopyranoside |
| f8 | 6.76 | 263, 341 | 287[A+H]^+^，475[M+H]^+^ | 284[A-2H] ^−^，473[M-H] ^−^ | kaempferol 3-(3”-acetylrhamnoside) |
| f9 | 7.04 | 257, 348 | 319[A+H]^+^, 549[M+H]^+^ | 316[A-2H] ^−^, 547[M-H] ^−^ | myricetin 3-*O*-α-L-(3”-*O*-malonyl)-rhamnopyranoside |
| f10 | 7.32 | 266, 354 | 319[A+H]^+^, 549[M+H-152]^+^, 701[M+H]^+^ | 316[A-2H] ^−^, 547[M-H-152] ^−^, 699[M-H] ^−^ | myricetin 3-*O*-(2”-*O*-galloyl-6”-*O*-malonyl-β-galactopyranoside) |
| f11 | 7.59 | 254, 347 | 303[A+H]^+^，533[M+H]^+^ | 300[A-2H] ^−^，531[M-H] ^−^ | quercetin 3-*O*-α-L-(3”-*O*-malonyl)-rhamnopyranoside |
| f12 | 7.83 | 264, 341 | 287[A+H]^+^，517[M+H]^+^ | 284[A-2H] ^−^，515[M-H] ^−^ | kaempferol 3-*O*-α-L-(3”-*O*-malonyl)-rhamnopyranoside |
