# Supplementary material for: Transcriptome sequencing and metabolite analysis for revealing the blue flower formation in waterlily
Source: BMC Genomics. 2016 Nov 9;17:897. doi: 10.1186/s12864-016-3226-9 (PMC5101690; doi:10.1186/s12864-016-3226-9)
Supplement: Additional file 3: Table S3. — Quantitative analysis of flavonoids in flower petals of Nymphaea ‘King of Siam’. (DOCX 18 kb) [file 12864_2016_3226_MOESM3_ESM.docx]

**Additional Table S3. Quantitative analysis of flavonoids in flower petals of *Nymphaea* ‘King of Siam’.**

| Sample | Content (mg 100 g^-1^ FW) | | | | | |
| --- | --- | --- | --- | --- | --- | --- |
|  | S1 | S2 | S3 | S4 | S5 | S6 |
| a1 | ND | 0.95±0.02 | 2.21±0.18 | 2.30±0.18 | 0.60±0.09 | 0.49±0.13 |
| a2 | ND | 1.35±0.01 | 5.63±0.08 | 8.91±0.14 | 2.16±0.06 | 0.23±0.02 |
| a3 | ND | 2.82±0.05 | 7.36±0.34 | 6.59±0.09 | 0.76±0.08 | 0.42±0.10 |
| a4 | ND | 4.82±0.12 | 35.20±0.51 | 65.35±0.85 | 13.17±0.85 | 2.58±0.21 |
| TA | ND | 9.94±0.18 | 50.39±0.38 | 83.16±0.71 | 16.69±0.95 | 3.73±0.45 |
| f1 | 4.29±0.07 | 7.23±0.05 | 8.69±0.48 | 9.78±0.34 | 4.90±0.48 | 2.64±0.34 |
| f2 | 0.96±0.02 | 4.71±0.07 | 18.49±1.13 | 38.36±0.99 | 58.71±3.48 | 62.25±5.76 |
| f3 | 9.72±0.80 | 8.90±0.48 | 13.19±0.77 | 16.70±0.58 | 10.79±0.63 | 7.69±0.61 |
| f4 | 0.29±0.01 | 0.34±0.04 | 0.95±0.07 | 1.76±0.05 | 3.10±0.19 | 4.66±0.57 |
| f5 | 0.35±0.01 | 0.58±0.05 | 0.93±0.07 | 4.28±0.87 | 160.50±10.20 | 149.53±12.60 |
| f6 | 0.02±0.00 | 0.13±0.00 | 0.64±0.03 | 3.02±0.05 | 27.58±1.85 | 21.61±1.87 |
| f7 | ND | ND | ND | ND | 25.45±1.82 | 28.96±2.48 |
| f8 | ND | ND | ND | ND | 5.47±0.40 | 5.30±1.16 |
| f9 | 0.78±0.00 | 2.88±0.03 | 9.79±0.10 | 33.64±0.52 | 263.10±14.72 | 172.45±12.17 |
| f10 | ND | ND | ND | 0.29±0.00 | 4.41±0.31 | 2.08±0.15 |
| f11 | ND | ND | ND | 0.35±0.01 | 18.29±1.30 | 13.18±1.00 |
| f12 | ND | ND | ND | 0.05±0.00 | 3.22±0.22 | 2.79±0.21 |
| TF | 16.41±0.88 | 24.77±0.42 | 52.67±2.29 | 108.23±1.72 | 585.53±35.35 | 473.71±37.72 |

ND means not detected this compound in flower petals.
