# Supplementary material for: Transcriptome sequencing and metabolite analysis for revealing the blue flower formation in waterlily
Source: BMC Genomics. 2016 Nov 9;17:897. doi: 10.1186/s12864-016-3226-9 (PMC5101690; doi:10.1186/s12864-016-3226-9)
Supplement: Additional file 4: Table S4. — Output statistics of RNA-Seq of Nymphaea ‘King of Siam’. (DOCX 17 kb) [file 12864_2016_3226_MOESM4_ESM.docx]

**Additional Table S4. Output statistics of RNA-Seq of *Nymphaea* ‘King of Siam’**

| **Samples** | **Total Reads** | **Average Length (bp)** | **Total Bases (Gb)** | **GC Percentage** | **Q30 Percentage** | **Total Bases** |
| --- | --- | --- | --- | --- | --- | --- |
| S1-1 | 24,496,349 | 125 | 6.17 | 46.56% | 90.87% | 6,171,559,592 |
| S1-2 | 23,391,214 | 125 | 5.89 | 46.92% | 90.93% | 5,893,564,049 |
| S1-3 | 22,412,846 | 125 | 5.65 | 46.70% | 90.55% | 5,645,659,642 |
| S3-1 | 24,894,909 | 125 | 6.27 | 46.83% | 90.90% | 6,272,307,795 |
| S3-2 | 25,783,914 | 125 | 6.5 | 47.20% | 90.78% | 6,496,071,901 |
| S3-3 | 25,845,223 | 125 | 6.51 | 46.91% | 90.84% | 6,510,430,395 |

Q30 percentage indicates the percentage of sequences with sequenceing error rate lower than 1‰
