# Supplementary figures and images for: Transcriptome sequencing and metabolite analysis for revealing the blue flower formation in waterlily
Source: BMC Genomics. 2016 Nov 9;17:897. doi: 10.1186/s12864-016-3226-9 (PMC5101690; doi:10.1186/s12864-016-3226-9)

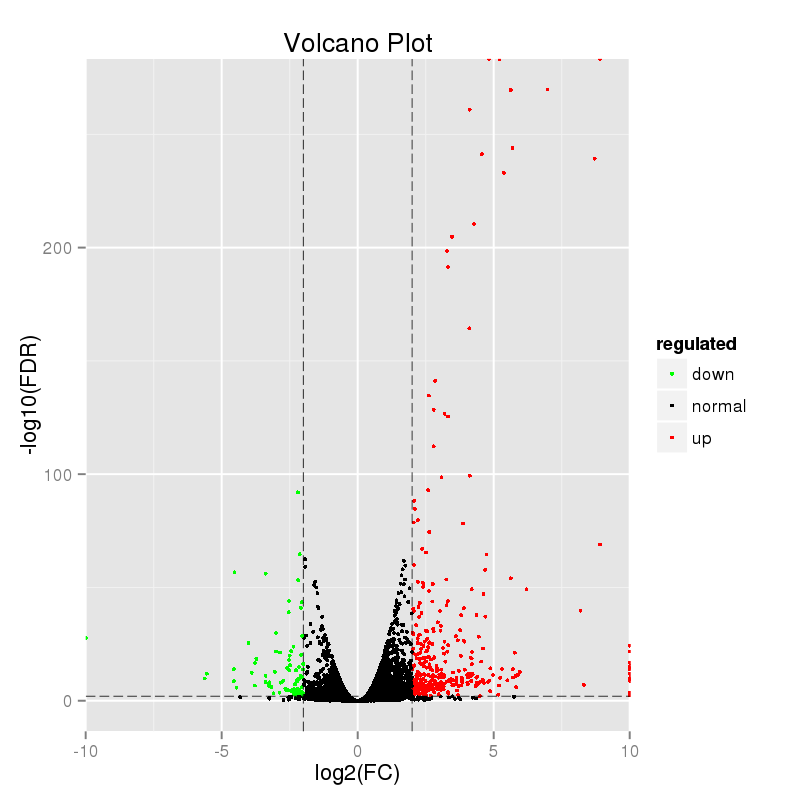

Supplement: Additional file 5: Figure S1. — Volcano plot between S1 and S3. (PNG 28 kb) [file 12864_2016_3226_MOESM5_ESM.png]

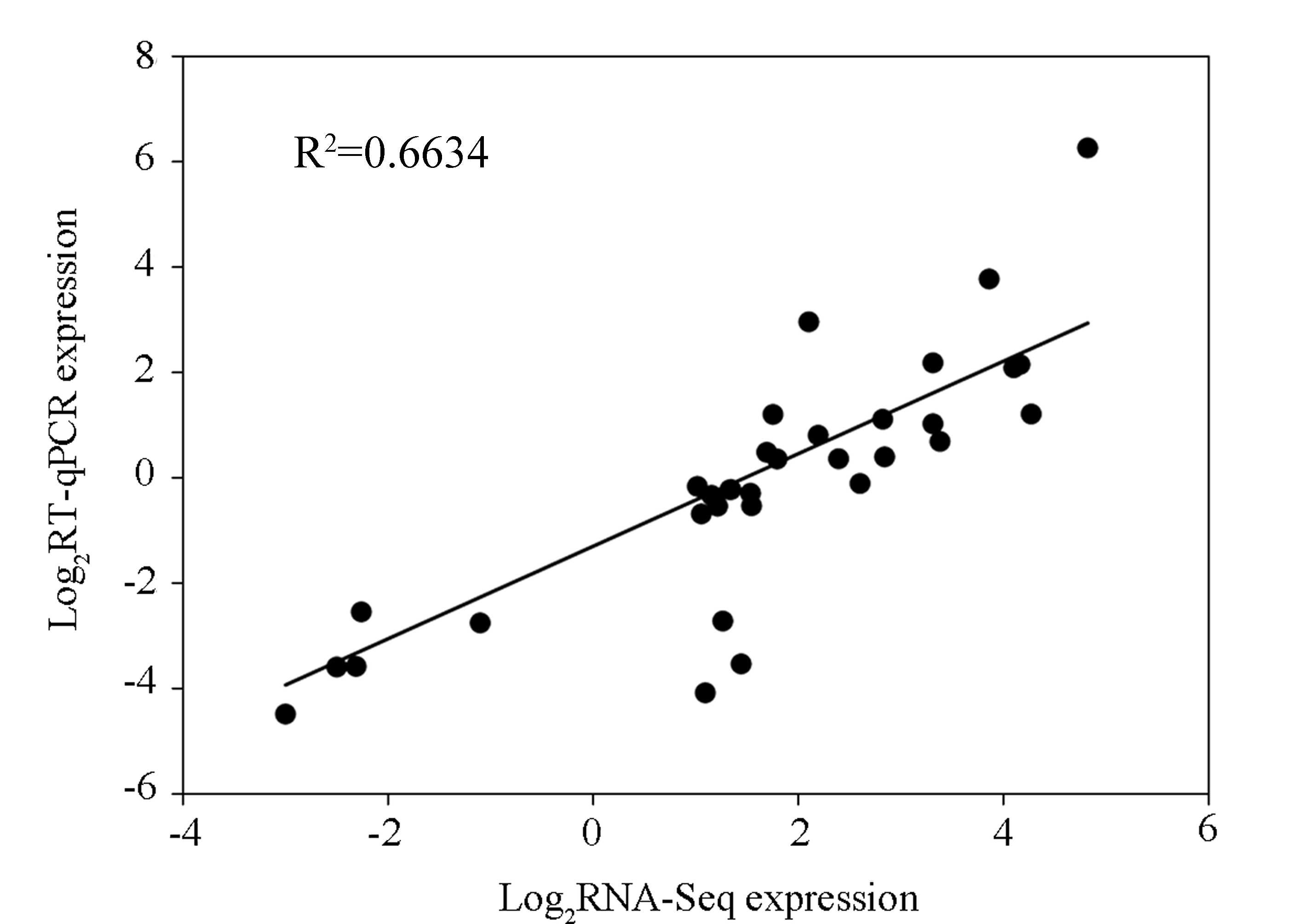

Supplement: Additional file 7: Figure S2. — Correlation of gene expression results respectively obtained by two methods (RT-qPCR analysis and RNA-Seq) for color-related genes in S1 and S3. (TIF 1641 kb) [file 12864_2016_3226_MOESM7_ESM.tif]
