# Supplementary material for: Transcriptome sequencing and metabolite analysis for revealing the blue flower formation in waterlily
Source: BMC Genomics. 2016 Nov 9;17:897. doi: 10.1186/s12864-016-3226-9 (PMC5101690; doi:10.1186/s12864-016-3226-9)
Supplement: Additional file 6: Table S5. — List of pathway enriched differentially expressed genes in Nymphaea ‘King of Siam’. (DOCX 16 kb) [file 12864_2016_3226_MOESM6_ESM.docx]

**Additional Table S5. List of pathway enriched differentially expressed genes in *Nymphaea* ‘King of Siam’.**

| No. | Pathway | Pathway ID | DEGs | Enrichment factor | Correct p-value |
| --- | --- | --- | --- | --- | --- |
| 1 | Phenylalanine metabolism | ko00360 | 20 | 0.12 | 0 |
| 2 | Phenylpropanoid biosynthesis | ko00940 | 24 | 0.12 | 0 |
| 3 | Flavonoid biosynthesis | ko00941 | 21 | 0.07 | 0 |
| 4 | Stilbenoid, diarylheptanoid and gingerol biosynthesis | ko00945 | 10 | 0.07 | 6.61E-09 |
