# Supplementary material for: Transcriptome sequencing and metabolite analysis for revealing the blue flower formation in waterlily
Source: BMC Genomics. 2016 Nov 9;17:897. doi: 10.1186/s12864-016-3226-9 (PMC5101690; doi:10.1186/s12864-016-3226-9)
Supplement: Additional file 8: Table S6. — List of UA3′GT in Nymphaea ‘King of Siam’. (DOCX 17 kb) [file 12864_2016_3226_MOESM8_ESM.docx]

**Additional Table S6. List of *UA3'GT* in *Nymphaea* ‘King of Siam’.**

| **Unigene** | **Annotation** | **FPKM (S1)** | **FPKM (S3)** | **Log_2_(S3/S1)** |
| --- | --- | --- | --- | --- |
|  |  |  |  |  |
| c55716.graph_co | Anthocyanin 3’-*O*-beta-glucosyltransferase-like (*Eucalyptus grandis*) | 12.345 | 12.16 | -0.02178361 |
| c65630.graph_co | Anthocyanin 3’-*O*-beta-glucosyltransferase-like (*Cicer arietinum*) | 9.59 | 8.87 | -0.11259671 |
| c68131.graph_co | Anthocyanin 3’-*O*-beta-glucosyltransferase-like (*Cicer arietinum*) | 1.175 | 2.26 | 0.943662016 |
| c70576.graph_co | Anthocyanin 3’-*O*-beta-glucosyltransferase-like (*Theobroma cacao*) | 1.045 | 1.005 | -0.05630744 |
| c70576.graph_c1 | Anthocyanin 3’-*O*-beta-glucosyltransferase-like (*Cicer arietinum*) | 2.085 | 1.51 | -0.46549883 |
| c74505.graph_co | Anthocyanin 3’-*O*-beta-glucosyltransferase-like (*Cicer arietinum*) | 29.42 | 32.69 | 0.152052131 |
